# Shifting Substance Use Landscapes: Changing Treatment Admission Trends in New York State, 2019–2023

**DOI:** 10.1192/j.eurpsy.2026.10203

**Published:** 2026-07-31

**Authors:** S. Jaka, C. W. Wong, J. Singh, S. Kaur, N. Thakurathi

**Affiliations:** Psychiatry and Behavioral Health Sciences, Nassau Univeristy Medical center, East Meadow, United States

## Abstract

**Introduction:**

Substance use treatment admissions provide key insight into evolving public health needs. In New York State, pandemic-era disruptions and longer-term demographic patterns have shaped treatment utilization. A multi-dimensional analysis of admissions by year, sex, age, and substance type can inform planning and resource allocation.

**Objectives:**

To examine trends in substance use treatment admissions in New York State from 2019 to 2023, with a focus on changes by year, sex, age group, and primary substance type, in order to identify persistent disparities and emerging patterns that can inform future public health planning and resource allocation.

**Methods:**

We analyzed SAMHSA’s CBHSQ Quick Statistics data for New York (2019–2023). Annual totals and proportional distributions were extracted across sex (male, female, unknown), age (12–17, 18–25, 26–34, 35–44, 45–54, 55–64, ≥65), and primary substance categories (alcohol, heroin, marijuana/hashish, cocaine, methamphetamine, other opiates). Trends were summarized descriptively and evaluated with linear regression to estimate direction and magnitude of change.

**Results:**

Total treatment admissions declined from 271,148 in 2019 to 183,659 in 2022 (–32.3%), stabilizing in 2023 at 184,552. Year-over-year changes were –25.4%, –6.2%, –3.2%, and +0.5% respectively. A linear trend indicated an average decline of ~19,000 admissions per year, projecting ~176,000 admissions in 2024. Sex composition was stable, with male patients comprising 72–73% of admissions annually. Age composition showed consistent concentration in 18–34 and 35–54 year-olds, with the lowest share among adolescents (12–17). Substance-specific analysis revealed that alcohol remained the single largest category, while heroin admissions declined steadily, and marijuana-related admissions were disproportionately concentrated among younger adults (18–25). Methamphetamine admissions, though a smaller proportion overall, showed relative growth, particularly among younger and mid-age groups.

**Conclusions:**

Between 2019 and 2023, New York state saw a marked decline in substance use treatment admissions followed by stabilization, yet demographic and substance-specific patterns remained relatively consistent. Men and adults aged 18–54 accounted for the majority of admissions. Alcohol and heroin dominated overall treatment demand, but shifting age and sex patterns for marijuana and methamphetamine signal areas requiring close monitoring. These findings underscore the need for sustained, targeted interventions that consider both persistent demographic disparities and emerging substance-specific trends

**Disclosure of Interest:**

None Declared

